# Targeting epidermal growth factor receptor to recruit newly generated neuroblasts in cortical brain injuries

**DOI:** 10.1186/s12967-023-04707-1

**Published:** 2023-11-30

**Authors:** Ricardo Gómez-Oliva, Noelia Geribaldi-Doldán, Samuel Domínguez-García, Ricardo Pardillo-Díaz, Sergio Martínez-Ortega, José M. Oliva-Montero, Patricia Pérez-García, Francisco J. García-Cózar, Juan P. Muñoz-Miranda, Ismael Sánchez-Gomar, Pedro Nunez-Abades, Carmen Castro

**Affiliations:** 1 Área de Fisiología, Facultad de Medicina, Universidad de Cádiz, Cádiz, Spain; 2 Instituto de Investigación e Innovación Biomédica de Cádiz, Cádiz, Spain; 3 Departamento de Anatomía y Embriología Humanas, Facultad de Medicina, Universidad de Cádiz, Cádiz, Spain; 4 Present Address: Department of Neuroscience, Karolinska Institutet, Biomedicum, Stockholm, Sweden; 5 Hospital Universitario Puerta del Mar, Cadiz, Spain; 6 Área de Inmunología, Universidad de Cádiz, Cádiz, Spain; 7 Servicios Centrales de Investigación Biomédica, Universidad de Cádiz, Cádiz, Spain; 8 Departamento de Fisiología, Universidad de Sevilla, Sevilla, Spain

**Keywords:** Transforming growth factor alpha, Adult neurogenesis, Brain injuries, Neuroblast migration, Neuroregeneration

## Abstract

**Background:**

Neurogenesis is stimulated in the subventricular zone (SVZ) of mice with cortical brain injuries. In most of these injuries, newly generated neuroblasts attempt to migrate toward the injury, accumulating within the *corpus callosum* not reaching the perilesional area.

**Methods:**

We use a murine model of mechanical cortical brain injury, in which we perform unilateral cortical injuries in the primary motor cortex of adult male mice. We study neurogenesis in the SVZ and perilesional area at 7 and 14 dpi as well as the expression and concentration of the signaling molecule transforming growth factor alpha (TGF-α) and its receptor the epidermal growth factor (EGFR). We use the EGFR inhibitor Afatinib to promote neurogenesis in brain injuries.

**Results:**

We show that microglial cells that emerge within the injured area and the SVZ in response to the injury express high levels of TGF-α leading to elevated concentrations of TGF-α in the cerebrospinal fluid. Thus, the number of neuroblasts in the SVZ increases in response to the injury, a large number of these neuroblasts remain immature and proliferate expressing the epidermal growth factor receptor (EGFR) and the proliferation marker Ki67. Restraining TGF-α release with a classical protein kinase C inhibitor reduces the number of these proliferative EGFR^+^ immature neuroblasts in the SVZ. In accordance, the inhibition of the TGF-α receptor, EGFR promotes migration of neuroblasts toward the injury leading to an elevated number of neuroblasts within the perilesional area.

**Conclusions:**

Our results indicate that in response to an injury, microglial cells activated within the injury and the SVZ release TGF-α, activating the EGFR present in the neuroblasts membrane inducing their proliferation, delaying maturation and negatively regulating migration. The inactivation of this signaling pathway stimulates neuroblast migration toward the injury and enhances the quantity of neuroblasts within the injured area. These results suggest that these proteins may be used as target molecules to regenerate brain injuries.

**Supplementary Information:**

The online version contains supplementary material available at 10.1186/s12967-023-04707-1.

## Introduction

The discovery of neurogenesis in specific regions of the adult brain, revealing its capacity to generate new neurons from neural stem cells (NSC), has represented a breakthrough in the understanding of brain plasticity and repair [1, 2]. Furthermore, unearthing the fact that neurogenesis is positively regulated in specific brain regions in response to damage has raised much expectation over the past few years about the opportunity of developing future therapies aimed to regenerate the damaged brain [3]. Physiologically, neurons are replaced in specific regions of the adult brain. One of these regions is the olfactory bulb (OB), which continuously integrates neuroblasts generated in the subventricular zone (SVZ) [2]. OB neurogenesis is initiated in the SVZ upon activation of neural stem cells (NSC) that enter the cell cycle to generate highly proliferative transit amplifying cells (TAC). These undifferentiated progenitors give rise to neuroblasts. Neuroblasts may undergo cell divisions while they migrate to the OB and differentiate into mature neurons [4–6]. This migration of SVZ neuroblasts toward the OB is tightly regulated by signals within the SVZ niche and the OB [7].

In response to injuries (traumatic, ischemic or other kinds) NSC are activated within the SVZ [8–10]. As a result, a higher number of neuroblasts can be found in the SVZ of mice with cortical brain injuries [11]. Interestingly, in response to severe ischemic injuries, a subset of these neuroblasts alter their physiological migration route toward the OB and attempt to migrate toward the damaged region [12–15]. However, neuroblast migration from the SVZ to a non-extensive focal cortical injury rarely occurs [16]. SVZ cells have been observed migrating toward brain injuries only in specific injured regions such as the striatum and few reports show migration toward the injured cortex in response to extensive ischemic injuries [17]. In most cases, the majority of the cells that initiate a migratory pathway toward the injury stay within the *corpus callosum* (CC), barely reaching the perilesional area [16, 18–20]. Nevertheless, accumulating evidence suggest that additional mechanisms may potentially lead to neuroblast enrichment in injured cortical areas. Proliferating cells have been observed in the injured cortex of mice, which may be manipulated to generate new neurons [21, 22]. However, despite the existence of these mechanisms, neuronal replacement in cortical injuries is variable and very limited. It depends on the extent of the injury and on the type of injury[10, 12, 23–25]. This mainly occurs because a gliogenic/non-neurogenic environment is generated within the injured tissue, that leads the fate of neural progenitors into glial cells [26]. This environment, probably generated by activated astrocytes, microglial cells, oligodendrocyte progenitors, and fibroblasts [27, 28] contributes to the formation of the glial scar. Cells forming the glial scar secrete a complex extracellular matrix that prevents neuronal migration towards the injury [29–31]. Particularly, inflammatory cues released by immune microglial cells in response to the damage [32] may be one of the main factors that lead to the inefficacy of the cells derived from the SVZ neurogenic niche to repair cortical brain injuries.

Thus, understanding the signaling molecules that determine the fate and of SVZ progenitors and the final destination of SVZ-derived neuroblasts in response to cortical injuries might highlight the role of molecules involved in cortical injury repair. In here, we have studied the role of transforming growth factor alpha (TGF-α) and its receptor, the epidermal growth factor receptor (EGFR). TGF-α is a signaling molecule previously reported as an inhibitor of neuroblast migration from the SVZ toward the OB [33, 34]. TGF-α is a member of the epidermal growth factor (EGF) family of ligands, structurally homologous to EGF that binds to EGFR [35]. This receptor is expressed in activated NSC and TAC, as well as in immature neuroblasts. The activity of this receptor and its associated pathways stimulate the proliferation of these cells [36–38]. TGF-α is synthesized as a membrane bound pro-ligand and released to the extracellular medium after the shedding of the soluble ligand in a reaction catalyzed by the metalloprotease ADAM17 and facilitated by kinases of the protein kinase C (PKC) family, particularly the classical PKCα [39, 40]. We hypothesize that mechanical cortical injuries upregulate the expression of TGF-α in the injured cortex and the SVZ, leading to the release of higher levels of this ligand. This stimulates the generation of neuroblasts and promotes their proliferation while it impairs neuroblast maturation and migration. Therefore, the inhibition of EGFR after a cortical injury may result in the migration of SVZ cells toward the injury resulting in neuroblast enrichment within the injured area. Since the use of EGFR inhibitor drugs has been approved to treat diseases such as non-small cell lung cancer (NSCLC), testing the effect of EGFR inhibitors may be useful to identify pharmacological treatments to regenerate cortical brain injuries.

## Methods

### Materials

Most products were purchased from Merck Life Science. The classical PKC inhibitor Gö6976 was purchased from Calbiochem (Millipore, Billerica, MA, USA). The classical PKC inhibitor was dissolved in dimethyl sulfoxide (DMSO) and diluted to a final concentration of 0.16 μM in phosphate-buffered saline (PBS) before the administration. The EGFR inhibitor Afatinib (MedChemExpress, USA) was dissolved in DMSO and diluted to a final concentration of 1 μM in PBS.

### Animal subjects

CD1 male mice were used throughout this study. Animals were housed under controlled conditions of temperature (21–23 °C) and light (LD 12:12) with free access to food (AO4 standard maintenance diet, SAFE, Épinay-sur-Orge, France) and water. Care and handling of animals were performed according to the Guidelines of the European Union Council (2010/63/EU), and the Spanish regulations (65/2012 and RD53/2013) for the use of laboratory animals. All studies involving animals are reported in accordance with the ARRIVE guidelines for reporting experiments involving animals [41, 42].

The number of animals used in each experiment was determined based on previous studies [43–45]. Adult male mice were randomized during the first week after birth by cross-fostering and used when they became two months old. The protocol used has been authorized by the Ethics Committee of the “Consejería de Agricultura, Ganadería, Pesca y Desarrollo Sostenible de la Junta de Andalucía”, in Spain with the approval numbers 04/03/2020/033 and 10/03/2020/039.

### Experimental design

Mice received controlled mechanical injuries in the primary motor cortex while anesthesized with a cocktail of 100 mg/kg ketamine and 20 mg/kg xylazine. For the migration studies, mice were injected with lentiviral vectors prior to the injury in the same surgical act. Once injured, the different treatments were administered intranasally as we describe in the paragraphs below. Upon the completion of the treatments, mice were anesthesized with a cocktail of 100 mg/kg ketamine and 20 mg/kg xylazine and CSF was extracted as explained below. Then, a dose of Dolethal^®^ (Ventoquinol, Lure, France) containing a lethal 50 mg dose of pentobarbital to euthanized the animals was applied followed by either brain perfusion (for histological postmortem studies) or brain extraction (for molecular biology studies). See description of the different procedures below.

### Mechanical cortical brain lesions

Controlled unilateral mechanical cortical brain injuries were performed in the primary motor cortex of the right brain hemisphere of anesthetized mice. A cocktail of 100 mg/kg ketamine and 20 mg/kg xylazine was used as anesthetic and injected intraperitoneally to mice (n = 5–6 per group as indicated in figure legends). Using a stereotaxic frame (Kopf Instrument), mice were craniotomized with a mechanical drill. Thereafter, a controlled mechanical lesion was performed in the underlying primary motor cortex using a manual drill (0.7 mm diameter) at 1.1 mm rostral and 1.5 mm lateral from Bregma. This drill was allowed to penetrate 1 mm below the bone surface. Mice were injured and placed into a controlled cage for 7 or 14 days post injury (dpi) depending on the treatment and experimental design. Lesions were performed unilaterally; the injured hemisphere was considered the ipsilateral side, while the intact hemisphere was considered the contralateral hemisphere and was used as a control. This procedure was previously stablished by our research group and has been used elsewhere [11, 18–20].

### Intranasal administration

The classical PKC inhibitor (Gö6976) and Afatinib were delivered intranasally as previously described while the animal was placed in a standing position with an extended neck [46, 47]. 18 μL of 1 μM Afaitinib, 0.16 μM Gö6976 or diluent (vehicle, containing 0.4% DMSO) was delivered over both nasal cavities alternating 3 μL/each using a micropipette. To ensure all fluid was inhaled, mice were maintained in the mentioned stained position for 10 additional seconds. Mice were coded, treatment (control or treatment) was assigned randomly to code numbers and applied. In addition, blind quantifications were performed.

### Cerebrospinal fluid extraction

Cerebrospinal fluid (CSF) collection was performed as described by Lim et al. [48]. Mice were anesthetized as described above and placed prone on the stereotaxic instrument. Muscles were moved to the side and dura mater over the cisterna magna was exposed. A capillary tube was placed and inserted into the *cisterna magna* through the *dura mater*, lateral to the *arteria dorsalis spinalis*. Finally, the CSF was collected through the capillary.

### Concentration of TGF-α in CSF

TGF-α was measured in the CSF using commercial ELISA kits, MBS2508394, (MyBioSourse, Inc, San Diego, CA), following the manufacturer’s instructions. CSF was centrifugated for 20 min at 1000xg and 4 °C; then supernatant was collected. Blanks (diluent only) were included in each independent determination. Blanks were subtracted from measurements before comparisons were made.

### RNA isolation, reverse transcription and real-time quantitative PCR (RT-qPCR)

For RT-qPCR analysis, RNA was isolated from the SVZ; intact SVZ were processed for RNA extraction using the TRIzol^™^ (Cat. 15,596,026, Invitrogen, Carlsbad, CA, USA), separation method, following the manufacturer’s instructions and resuspended in purified nuclease-free water. RNA was quantified using a BioTek’s Synergy^™^ Mx fluorimeter (BioTek Instruments, Inc, Winooski, VT, USA). cDNA was prepared from 500 ng RNA using iScript™ cDNA Synthesis Kit (Cat.1708890, Bio-Rad Laboratories Inc, Hercules, CA, USA) on a Techne Genius thermal cycler (Techne Ltd., Cambridge, UK). The 15 μl RT-qPCR reaction mix contained 7.5 μl 2X iTaq^™^ Universal SYBR^®^ Green Supermix (Cat. 1,725,122, Bio-Rad Laboratories Inc, Hercules, CA, USA), 10 nmol of both the forward and the reverse primers, and 1 µl of the sample. The PCR thermal profile included 40 cycles of denaturation at 95°C for 10 s, an annealing temperature according to each set of primers for 15 s, and extension at 72°C for 20 s, followed by a melting curve analysis. Each sample was analyzed in triplicate. The mRNA level of rRNA18S was used as internal control. Relative quantification values of mRNA expression were calculated as 2^–ΔΔCt^ (Livak Method). Oligonucleotides primers used in this study were designed by BLAST and were obtained from Merck (Madrid, Spain). Primer sequences (5´-3´) for detecting expression of mouse mRNA were the following: for TGF-α, FW: CCAGATTCCCACACTCAGT, RW: GGAGGTCTGCATGCTCACA; for EGFR, FW: CCAGACAGACGACGGGTCA, RW: GCTCTGGCTCTCCGGGATTA.

### Western blot

Tissue was homogenized using a commercial lysis buffer (Cell Signaling, USA) supplemented with a protease and phosphatase inhibitor cocktail (Sigma, USA). The homogenates were sonicated and centrifuged at 4° C for 5 min at 16,000 x g. Supernatants were collected and protein concentration was measured using the Pierce BCA Protein Assay Kit (Thermo Scientific, Waltham, MA, USA). Equal amounts (30 µg) of total protein from each cellular extract were subjected to SDS-PAGE and western blotting. Proteins were separated on gradient 4–15% precast polyacrylamide gels (Mini-PROTEAN TGX Stain-Free Protein Gels, BioRad, USA), followed by electrophoretic transfer to PVDF membranes (Schleicher & Schuell, Keene, Netherlands). Membranes were then soaked in blocking buffer (Invitrogen, Carlsbad, CA, USA) for 30 min and incubated overnight at 4 °C with primary anti TGF-α antibody (see Additional file 1: Table S1). Membranes were washed and signal was detected using commercial kits (Western Breeze, Invitrogen, Carlsbad CA, USA) containing either anti-rabbit or anti-mouse secondary antibodies conjugated to alkaline phosphatase, plus the corresponding chemiluminescent substrate following manufacturer’s instructions. Membranes were developed using Chemidoc Touch Imaging System 732BR1030 (BioRad, USA). Quantification of OD was done using FIJI software.

### SVZ lentiviral transductions and Afatinib treatment

Mice were mechanically injured in the primary motor cortex and were injected with a lentiviral vector expressing ZsGreen in the lateral ventricle (LV). The lentiviral construct was produced by us as described in previous reports [19]. The virus titer of the lentivirus solution was 40 × 10^3^ TU/ml and 1 μL of the lentiviral solution was injected within the LV, to perform the lentivirus administration a small trepanation was made 0.8 mm lateral to Bregma and a needle of 100 μL Hamilton Neuros syringe (0.108 mm internal diameter) was introduced 2.4 mm below the brain surface. This lentivirus only induced the expression of ZsGreen in infected cells and accounted for any effect caused by transduction. The EGFR inhibitor Afatinib (1 μM) was administrated during 14 days after the injury and the lentiviral administration procedure.

### Brain processing and immunohistochemistry

At the end of treatments, mice were deeply anesthetized with Dolethal® (Ventoquinol, Lure, France) containing a lethal 50 mg dose of pentobarbital and perfused with 4% paraformaldehyde via ascending aorta. After perfusion, brains were sliced into 30 μm sections using a cryotome. Immunostaining was performed as previously described [18, 44, 49, 50]. See antibodies in Additional file 1: Tables S1, S2.

### Quantification of neurogenesis in brain sections

After perfusion, mouse brains were codded and blind quantification was performed. SVZ cells positive for the different markers were estimated as previously described [19]. Positive cells were counted throughout the entire lateral or laterodorsal walls of the lateral ventricles in every fifth section; analyzing 14–16 sections per brain under confocal microscopy (Zeiss LSM 900 Airyscan 2). The SVZ cell quantification was done in 30 μm thick serial coronal sections including, in the rostrocaudal axis in relation to Bregma, from + 1.54 to −0.94 mm. Confocal imaging was taken every 2 μm in the Z-plane using 20X or 63X objectives. Cell density was calculated for each section relative to the SVZ volume (mm^3^) and averaged for each animal as reported previously [51]. Both SVZ quantification was performed using the ImageJ software.

To analyze cell number in the perilesional area, 3–5 sections containing the cortical injury were selected. Positive cells of each marker were counted in each section including 200 μm-wide band of the adjacent injured border tissue. Cell density (number of cells counted divided by injured area and by section thickness) was calculated for each section, and averaged for each animal.

### Statistical analysis

All data and statistical analysis comply with the recommendations on experimental design and analysis in pharmacology [52]. The Student t-test for paired samples was used to compare ipsilateral vs. contralateral brain hemispheres. One-way ANOVA, followed by post-hoc Bonferroni test was used to compare the groups under study. IBM SPSS statistics 22 software was used for all statistical analysis. Differences were considered significant values of p < 0.05. Generally, sample size used in statistical analysis was n = 4–6, and was chosen based on previous works [19, 44].

## Results

### Expression of TGF-α is altered in the SVZ and injured cortex in response to a mechanical cortical injury

**Fig. 1:**
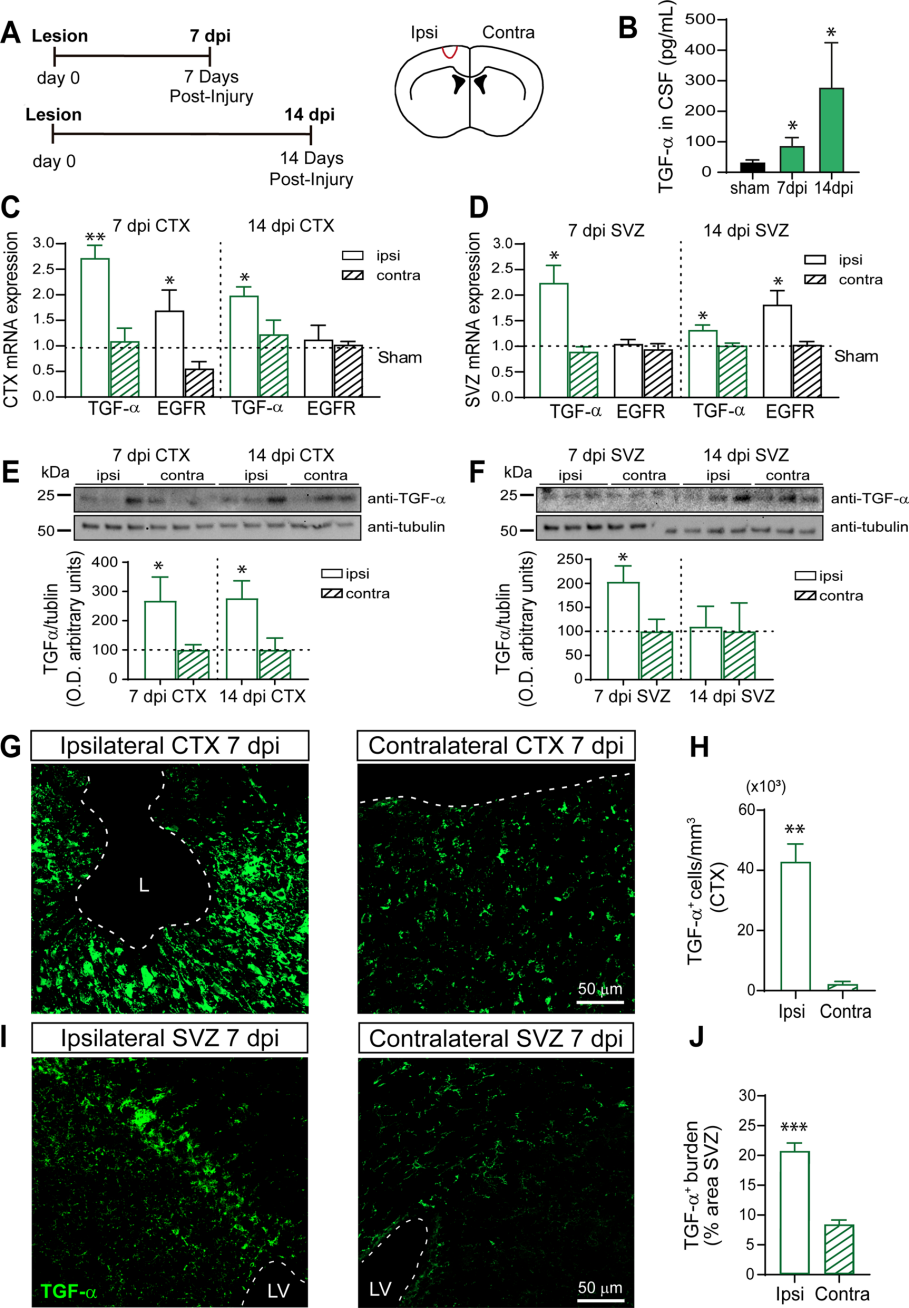
Elevated levels of TGF-α in the SVZ, injured cortex and CSF in response to a mechanical cortical injury.** A** Scheme of experimental procedures. **B** Mechanical cortical lesions were unilaterally performed in the adult mouse primary motor cortex. Mouse CSF was obtained 7 days post-injury (dpi). The graph shows TGF-α detection (pg/mL) in CSF at 7 dpi and in non-injured mice (sham). **C** Relative mRNA expression of TGF-α, and EGFR in the injured cortex (ipsi) and in non-injured cortex (contra) at 7 dpi and 14 dpi. mRNA expression was measured using RT-qPCR. **D** Relative mRNA expression of TGF-α and EGFR in the SVZ of the injured ipsilateral side (ipsi) and non-injured side (contra) 7 dpi and14 dpi. mRNA expression was measured using RT-qPCR. The data show the means ± SEM. (*p < 0,05, **p < 0,01, ipsilateral vs. contralateral) in one-way ANOVA, followed by posthoc Bonferroni test and Student’s t test for equal-variance paired samples. Data are the mean ± S.E.M; n = 4–6 animals per group. **E–F** Images of chemiluminescence signal of immunoblot detection of TGF-α and β-tubulin (loading control) in the cortex and SVZ of 7 dpi and 14 dpi mice. Integrated optical density quantification of images obtained from immunoblots: cortex and SVZ protein expression of TGF-α according to β-tubulin. **G** Representative confocal images showing the area surrounding the cortical lesion (ipsilateral) and the non-injured hemisphere (contralateral) processed for the immunodetection of TGF-α. The dotted line indicates the limit of the lesion (L) and the scale bar represents 50 μm. **H** Graph represents the number of TGF-α^+^ cells in the perilesional area compared to contralateral cortex. **I** Representative confocal images of adult mice SVZ bearing a unilateral cortical injury. Ipsilateral and contralateral SVZ were processed for the immunodetection of TGF-α. The dotted line indicates the limits of the lateral ventricles (LV). The scale bar represents 50 μm.** J** Graph shows TGF-α burden as a percentage of the total SVZ area in ipsilateral and contralateral SVZ. Statistical analysis: *p < 0.05 compared the ipsilateral site with the contralateral side in a Student’s t-test for equal-variance unpaired samples. Data are the mean ± S.E.M; n = 6 animals per group

Unilateral mechanical cortical injuries in the primary motor cortex of mice were performed and the levels of the mRNAs encoding TGF-α and EGFR were analyzed 7 and 14 dpi (Fig. 1 A) by RT-qPCR in the ipsilateral and contralateral SVZ and cortex (Fig. 1 C). In the injured cortex, the mRNA encoding TGF-α and EGFR increased by 3-and 1.5-fold respectively in the ipsilateral injured side in comparison with the contralateral side 7 dpi (Fig. 1 C). At 14 dpi, the elevated levels of TGF-α mRNA in the injured cortex remained elevated whereas EGFR levels declined to reach those found in the contralateral side and in control mice (Fig. 1 C). In the SVZ, the levels of TGF-α mRNA increased by twofold at 7 dpi and remained elevated at 14 dpi (Fig. 1 D). Interestingly, in the SVZ, no changes were observed in the expression of EGFR at 7 dpi (Fig. 1 D) but a twofold increase in EGFR mRNA was found at 14 dpi. The protein levels detected by western blot matched the mRNA expression (Fig. 1. E, F). However, elevated mRNA or protein levels do not necessarily need to lead to a higher concentration of TGF-α in the extracellular medium, thus we tested the concentration of soluble TGF-α in the CSF. In agreement, with the modulation of mRNA expression, the concentration of TGF-α in the CSF of injured mice at 7 dpi increased by nearly threefold (Fig. 1 B) and these elevated levels were maintained 14 dpi.

### Injury-induced increase in the number of TGF-α ^+^ microglial cells in the SVZ and cortex

**Fig. 2:**
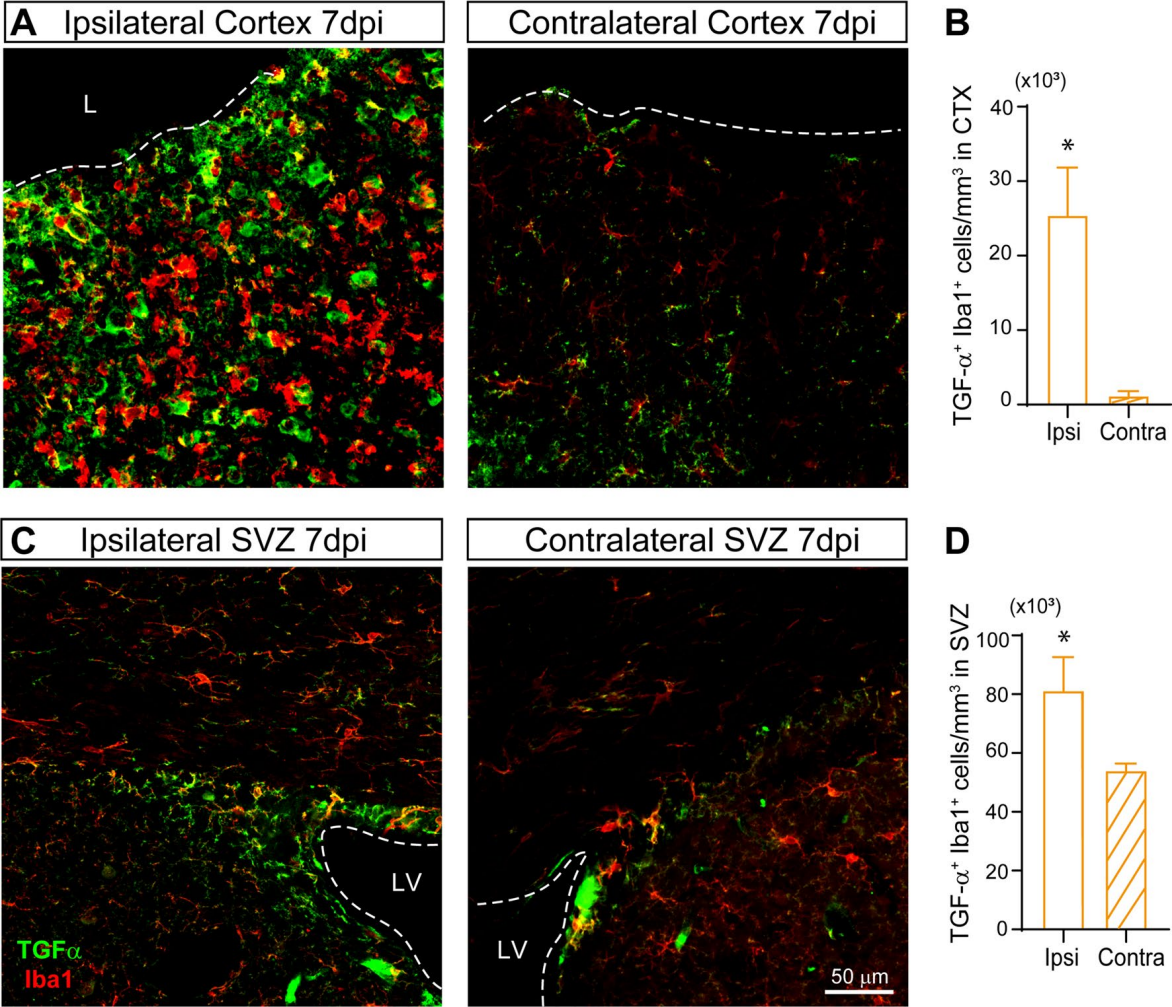
The number of TGF-α^+^ microglial cells increases after a cortical injury in the SVZ and perilesional area. Mechanical cortical lesions were unilaterally performed in the primary cortex of adult male mice. Mice were sacrificed 7 days post-injury (dpi) and processed for the immunodetection of TGF-α and the microglial marker Iba1. **A** Representative confocal images showing the area surrounding the cortical lesion (ipsilateral) and the non-injured hemisphere (contralateral) processed for the immunodetection of TGF-α and Iba1. The dotted line indicates the limit of the lesion (L) and the scale bar represents 50 μm**. B** Graph represents the number of TGF-α^+^-Iba1^+^ cells in the perilesional area compared to contralateral cortex. **C** Representative confocal images of adult mice SVZ bearing a unilateral cortical injury. Ipsilateral and contralateral SVZ were processed for the immunodetection of TGF-α and Iba1. The dotted line indicates the limits of the lateral ventricles (LV). The scale bar represents 50 μm. **D** Graph shows the number of TGF-α^+^-iba1^+^ cells in the ipsilateral and contralateral SVZ. Statistical analysis: * p < 0.05 compared the ipsilateral side with the contralateral in a Student´s t-test for equal-variance unpaired samples. Data are the mean ± S.E.M; n = 4–6 animals per group

In order to analyze if a higher number of cells expressed TGF-α, we studied the presence of TGF-α ^+^ cells in the SVZ and in the injured cortex of mice at 7 dpi. A fourfold increase in the number of cells labelled with TGF-α was found in the injured cortex (Fig. 1 E, F). Accordingly, a 2.5-fold increase in the TGF-α burden was found 7 dpi in the ipsilateral SVZ in comparison with the contralateral SVZ (Fig. 1 G, H). We next analyzed the phenotype of TGF-α ^+^ cells within the SVZ and cortex of injured mice. Interestingly, a dramatical increase in the number of Iba1^+^/TGF-α ^+^ was observed in the ipsilateral cortex of injured mice. A smaller but significant increase was also found in the SVZ. The posterior analysis of these results indicated that the phenotype of the cells expressing TGF-α in the injured cortex (Fig. 2 A, B) and the SVZ (Fig. 2 C, D) was mainly Iba1^+^ microglial cells.

### The number of immature SVZ neuroblasts increases in response to a cortical injury

**Fig. 3:**
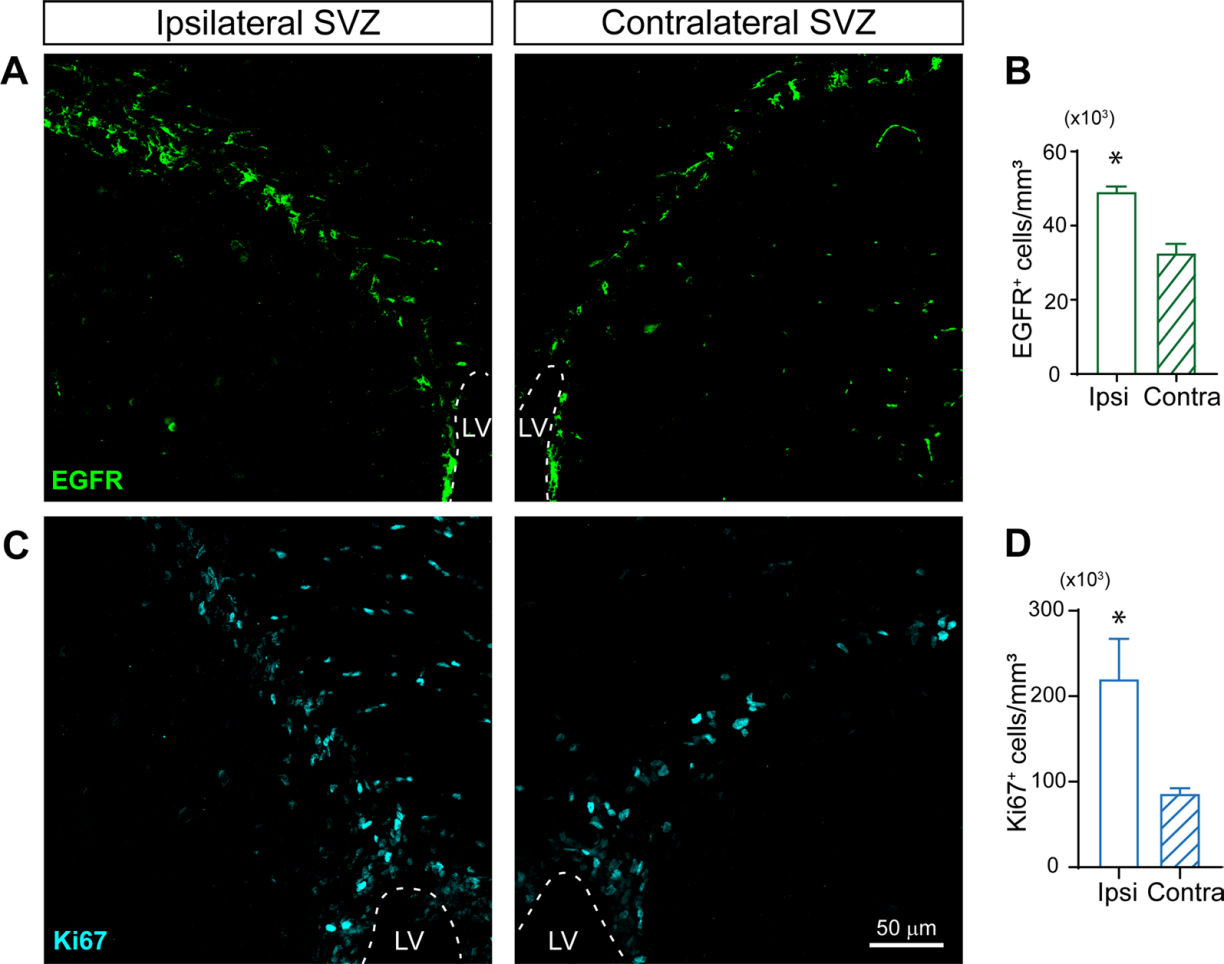
The number of proliferative cells increases in the SVZ after a cortical injury. Mechanical cortical lesions were unilaterally performed in the primary cortex of adult male mice. Mice were sacrificed at 7 days post-injury (dpi) and processed for the immunodetection of the epidermal growth factor receptor marker (EGFR), and the proliferation marker Ki67. **A** Representative confocal images of adult mouse SVZ bearing a unilateral cortical injury showing the immunodetection of EGFR. The dotted line indicates the limits of the lateral ventricles (LV). The scale bar represents 50 μm. **B** Graph shows the number of EGFR^+^ cells per mm^3^ in the ipsilateral and the contralateral SVZ. **C** Representative confocal images of adult mice SVZ bearing a unilateral cortical injury showing the immunodetection of the proliferation marker Ki67. **D** Graph shows the number of Ki67^+^ cells per mm^3^ in the ipsilateral and the contralateral SVZ. Statistical analysis: * p < 0.05 compared the ipsilateral with contralateral side using a Student´s t-test for equal-variance unpaired samples. Data are the mean ± S.E.M; n = 4–6 animals per group

**Fig. 4:**
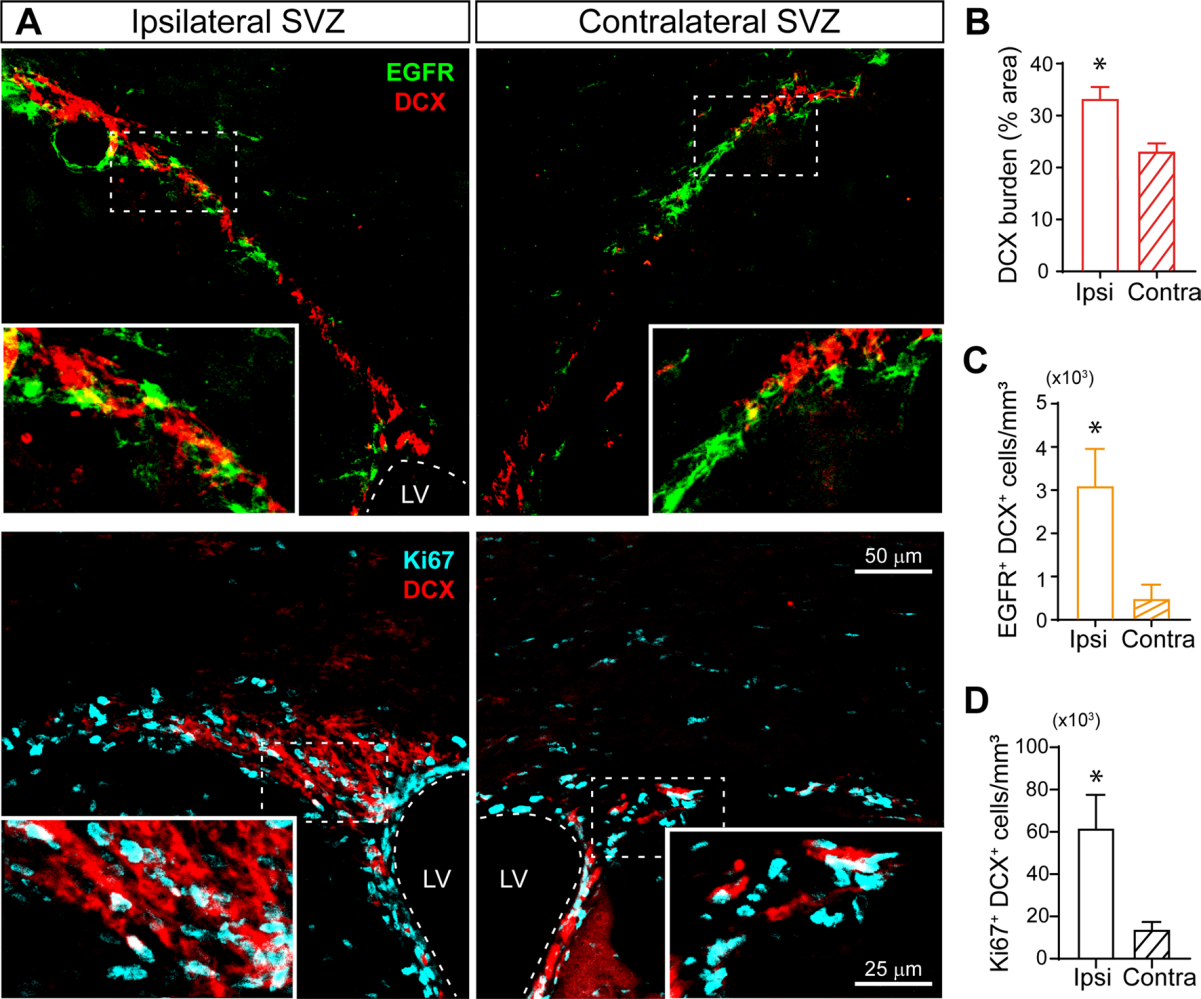
The number of immature SVZ neuroblasts increases in response to a cortical injury. **A** Representative confocal images of adult mice SVZ bearing a unilateral cortical injury. Mice were sacrificed at 7 days post injury (dpi) and processed for the immunodetection of the early neuroblast marker doublecortin (DCX), the epidermal growth factor receptor marker (EGFR) (upper panel), and of the proliferation marker Ki67 (lower panel). The dotted line indicates the limits of the lateral ventricles (LV). The scale bar represents 50 μm and 25 μm in the high magnification images. **B** Graph shows the burden of DCX^+^ cells as a percentage of the total SVZ area comparing the ipsilateral to the contralateral site. **C** Graph shows the number of EGFR^+^ cells that co-express the neuroblast marker DCX/mm^3^ in the ipsilateral and the contralateral SVZ. **D** Graph shows the number of Ki67^+^ cells that co-express the neuroblast marker DCX/mm^3^ in the ipsilateral and the contralateral SVZ. Statistical analysis: * p < 0.05 compared the ipsilateral with contralateral side using a Student´s t-test for equal-variance unpaired samples. Data are the mean ± S.E.M; n = 4–6 animals per group

Since TGF-α is a ligand of the EGFR receptor, we decided to analyzed the number of cells that expressed EGFR (EGFR^+^) in the SVZ of injured mice as well as the number of proliferative (Ki67^+^ cells). The number of cells expressing EGFR in the ipsilateral SVZ at 7 dpi increased by twofold (Fig. 3 A, B) and this increase was concomitant with a twofold enrichment in the number of Ki67^+^ cells (Fig. 3 C, D). In addition, the pool of neuroblasts (doublecortin expressing cell or DCX^+^ cells) increased by 1.5-fold in the ipsilateral SVZ at 7 dpi (Fig. 4 A, B), particularly, EGFR^+^ neuroblasts increased dramatically by sixfold in the ipsilateral SVZ (Fig. 4 A, C) and a twofold increase in the number of cycling Ki67^+^/DCX^+^ neuroblasts was found at 7 dpi (Fig. 4 A, D).

### Reduction of TGF-α release impairs the proliferative response in SVZ neuroblasts

**Fig. 5:**
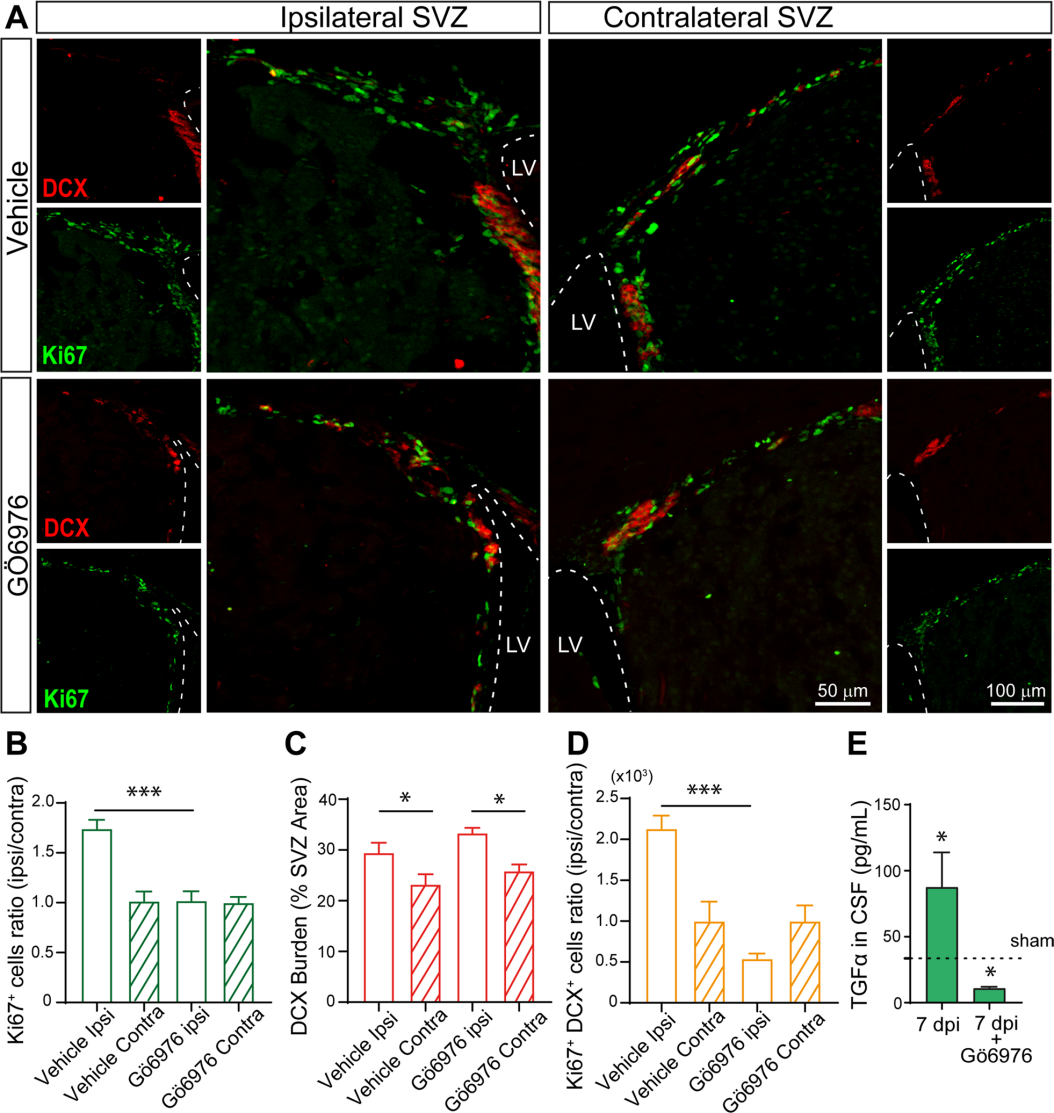
The proliferative response of SVZ neuroblasts to a cortical injury is inhibited in the presence of a classical PKC inhibitor that impairs TGF-α release. **A** Confocal microscopy images of the SVZ of adult mice bearing unilateral cortical injuries and administrated with the classical PKC inhibitor Gö6976 during 7 days. Dotted lines delineate lateral ventricles (LV) borders. Scale bar = 100 μm and 50 μm in the high magnification images. Mechanical cortical lesions were unilaterally performed in the primary cortex of adult male mice. Samples were processed for the immunodetection of the proliferation marker Ki67 and the early neuronal marker doublecortin (DCX). **B** Graph represents the average ratios obtained when dividing the number of Ki67^+^ cells in ipsilateral SVZ by the number of Ki67^+^ cells in the corresponding contralateral SVZ (considered as 1). Statistical analysis: * p < 0.05. Data are the mean ± S.E.M; n = 6 animals per group, using one-way ANOVA. **C.** Graph represents the DCX burden as a percentage of SVZ total area. **D** Graph represents the average ratios obtained when dividing the number of Ki67^+^-DCX^+^ cells in ipsilateral SVZ by the number of Ki67^+^-DCX^+^ cells in the corresponding contralateral SVZ (considered as 1). Statistical analysis: * p < 0.05. Data are the mean ± S.E.M; n = 6 animals per group, using one-way ANOVA. **E** Levels of TGF-α in the CSF of control and injured mice treated with vehicle or the cPKC inhibitor for 7 days after the injury were obtained after deep anesthesia following the experimental procedure detailed in materials and methods. The levels of TGF-α in non-injured mice (sham) were represented by a dotted line. Statistical analysis: * p < 0.05. Data are the mean ± S.E.M; n = 4–6 animals per group, using a Student´s t-test for equal-variance unpaired samples

In order to determine whether higher concentrations of TGF-α were responsible for the increased number of proliferating cells, particularly, proliferating neuroblasts, the release of TGF-α was then inhibited using the classical protein kinase C (cPKC) inhibitor (Gö6976). Evidences show that cPKC activation stimulates the ADAM17-mediated release of TGF-α in vitro [40] and therefore, the treatment of mice with this inhibitor is supposed to reduce TGF- α release. Mice were injured and treated for 7 days with intranasal administrations of the cPKC inhibitor Gö6976 or vehicle. The concentration of TGF-α in the CSF as well as the number of cycling cells and neuroblasts in the SVZ were analyzed at 7 dpi. As shown in Fig. 5, Gö6976 treatment reduces the concentration of TGF- α in the CSF of treated mice by sixfold (Fig. 5 E), concomitantly, the increase in the proportion of cycling cells in the ipsilateral SVZ of injured mice that was observed as a consequence of the injury, was not found upon treatment with Gö6976 (Fig. 5 A, B) and the proportion of proliferating neuroblasts not only was not elevated in the ipsilateral SVZ but it was reduced by twofold in comparison with the contralateral side (Fig. 5 A, D). The burden of DCX labelling was not affected by the use of the inhibitor (Fig. 5 A, C). These results suggested a role for TGF-α on stimulating the proliferation of neuroblasts and maintaining the immature phenotype of neuroblasts within the SVZ.

### Inhibition of EGFR facilitates neuroblasts enrichment within the injured area

**Fig. 6:**
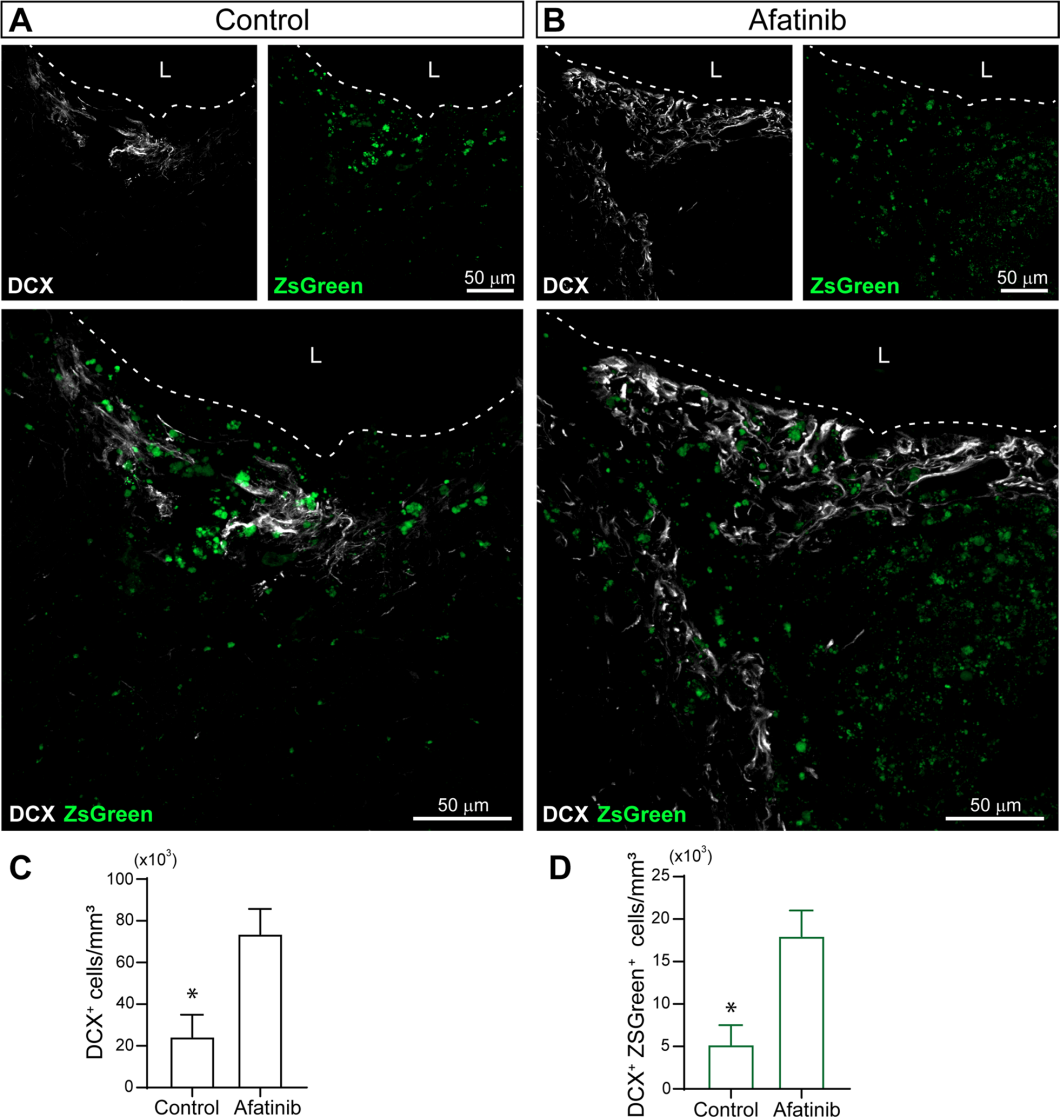
Inhibition of EGFR promotes migration of neuroblast and enrichment of neuroblasts within the injured area. Mice were mechanically injured in the primary motor cortex and were injected in the lateral ventricle (LV) with a control lentivirus that express the green fluorescent protein ZsGreen. Mice were treated for 14 days with intranasal administrations of the EGFR inhibitor Afatinib (1 μM) or vehicle. **A** Representative confocal images of the injured cortex of adult mice treated with vehicle and processed for the detection of DCX and ZsGreen. **B** Representative confocal images of the injured cortex of adult mice treated with Afatinib showing ZsGreen fluorescence and the immunodetection of DCX. The dotted line indicates the limit of the lesion (L) and the scale bar represents 50 μm**. C** Graph represents the number of DCX^+^ cells in the perilesional area comparing Afatinib treated mice with vehicle treated mice. **D** Graph represents the number of DCX^+^ cells that are also, ZsGreen^+^ cells. Statistical analysis: * p < 0.05. Data are the mean ± S.E.M; n = 4–6 animals per group, using a Student´s t-test for equal-variance unpaired samples

Then, in order to demonstrate the role of TGF-α on neuroblast migration, in another set of experiments, we injected a lentiviral vector, expressing the green fluorescent protein ZsGreen, in the lateral ventricle of adult mice. On the same day a mechanical injury was performed in the motor cortex. Then mice were treated with intranasal administrations of the EGFR inhibitor Afatinib for 14 days and mice were sacrificed at 14 dpi (Fig. 6 A-D). We observed that in control untreated mice, cells containing green fluorescence were found within the perilesional area, a small number of them being DCX^+^ cells. However, in mice treated with Afatinib, a fivefold increase in the number of DCX^+^/ZsGreen^+^ cells was found within the perilesional area. In the SVZ, no differences were observed in the DCX^+^ burden or the number of DCX^+^/ZsGreen^+^ cells (Additional file 1: Figure S1).

### Migration of neuroblasts toward the injury is induced by the inhibition of EGFR

**Fig. 7:**
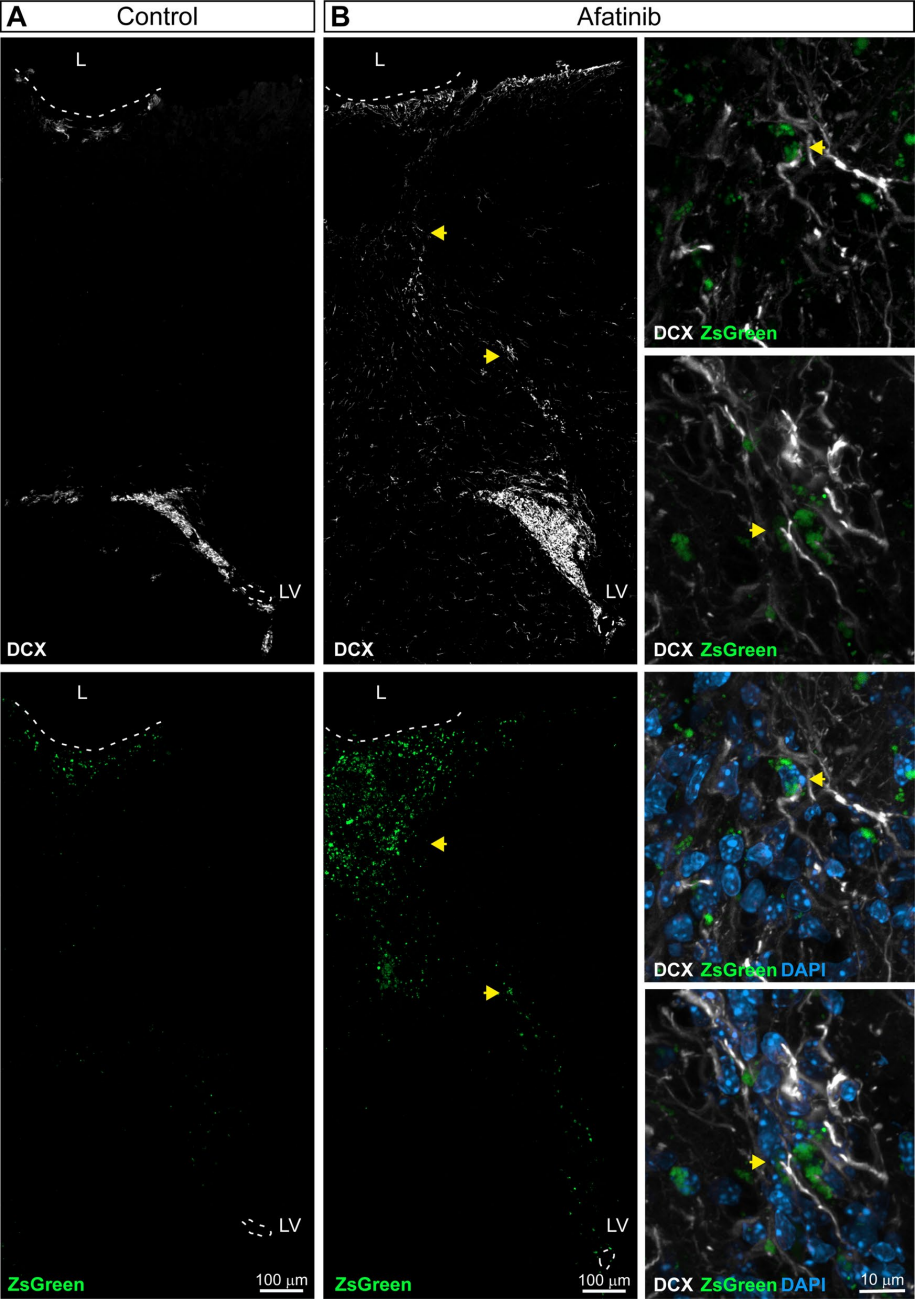
Inhibition of EGFR promotes migration of neuroblast to the injured area. Mice were mechanically injured in the primary motor cortex and were injected in the lateral ventricle (LV) with a lentivirus that expresses ZsGreen. Mice were treated for 14 days with the EGFR inhibitor Afatinib (1 μM) or vehicle. **A** Representative confocal images of the injured cortex of adult mice treated with vehicle showing ZsGreen fluorescence (upper image) and the immunodetection of DCX (lower image). **B** Representative confocal images the injured cortex of adult mice treated with Afatinib showing ZsGreen fluorescence (upper left) and the immunodetection of DCX (lower left). Magnification of the cells indicated with the yellow arrows shows ZsGreen and DCX expression (upper right images). Magnification of the cells indicated with the yellow arrows show ZsGreen, DCX and DAPI expression/signal (lower right images). The dotted line indicates the limit of the lesion (L) and the limits of the lateral ventricles (LV). Scale bar represents 100 μm and 10 μm

We next analyzed the number of DCX^+^/ZsGreen^+^ cells that were present in the region localized between the SVZ and the cortex. Interestingly, chains of migrating DCX^+^ cells, some of them showing green fluorescence, were observed at 14 dpi that had crossed the corpus callosum from the SVZ towards the cortex. These cells were only observed in mice treated with Afatinib (Fig. 7 B; see right panels for magnification) and not in control mice (Fig. 7 A). In order to understand the cellular mechanisms that were taking place we also analyzed the number of neuroblasts that had reached the ipsilateral and contralateral OB in the presence and absence of the treatment. In untreated mice, the area occupied by DCX^+^ cells in the OB was higher than that of the contralateral side. In Afatinib treated mice, the percentage of area occupied by DCX^+^ cells in the OB was as large as that found in the ipsilateral OB of non-treated mice and no differences were observed between the ipsilateral and contralateral side (Additional file 1: Figure S2). Additionally, no differences were observed on the number of ZsGreen^+^ cells or the number of DCX^+^/ZsGreen^+^ cells that migrated to the OB over the 7-day treatment period.

## Discussion

Brain injuries of different origins such as traumatic, ischemic or neurodegenerative stimulate neurogenesis in the SVZ resulting in a higher number of newly generated neuroblasts that attempt to migrate toward the injury [53] but do not reach the perilesional area [16, 19] not contributing to neuronal replacement and to the regeneration of the injured region. Failure of neuroblasts to migrate toward the injury has been reported to be a consequence of the presence or absence of signaling molecules that affect neuroblast migration [33, 54]. Understanding the cues that modulate neuroblast enrichment in the SVZ as well as the mechanisms that determine the migration of these cells toward the injury might lead to the generation of pharmacological drugs aimed to repair brain injuries. We show in here that injuries induce the expression of TGF-α in both the SVZ and the cortical injured area, resulting in elevated levels of TGF-α in the CSF. We show that TGF-α is mainly expressed in microglial cells that appear in both regions in response to the injury. As a consequence, neuroblasts remain in a prolonged immature state, characterized by cell cycle entrance, proliferation and the inability to differentiate and migrate toward the injury. Inhibition of the TGF-α receptor, EGFR, facilitates migration of neuroblasts from the SVZ toward the injury resulting in neuroblasts enrichment within the perilesional area.

Previous studies have demonstrated that brain damage caused by ischemia or by traumatic injury, stimulate neurogenesis in neurogenic niches or in the damaged area (reviewed in Nemirovich-Danchenko and Khodanovich 2019 [55]) depending on the extent of the injury and the affected area. Thus, the proliferation of neural stem cells and other progenitors is stimulated in the SVZ in response to ischemic injuries and some authors have shown that these progenitors migrate from the SVZ towards the damaged striatum producing neurons that integrate into existing circuits [12, 56, 57]. However, although other studies show the presence of a low number of newly formed neurons in the damaged cortex post injury [21], others have failed to demonstrate cortical neurogenesis post injury [11, 12, 19]. A few years ago, the work of Sundholm-peters et al. showed that in controlled cortical aspiration lesions a subset of neuroblasts emigrate from the SVZ dorsally into the corpus callosum. Interestingly, a higher number of neuroblasts were observed within the corpus callosum whereas no neuroblast reached the injured cortex [16]. Additional reports show that, upon application of BDNF [54], the inhibition of ADAM17 [19], or the treatment with PKC-activating compounds involved in neuregulin release, a significant number of neuroblasts can be found within the perilesional area of a cortical injury [20], demonstrating that modifying the environment created within the injury may facilitate the replacement of the damaged neurons by newly generated ones.

In here, we aimed to discover new mechanisms involved in the response of the brain to cortical injuries, we initially showed that an overexpression of TGF-α mRNA was found in the ipsilateral SVZ and cortex of injured mice within the perilesional area. This elevated expression was concomitant with an augmented concentration in soluble TGF-α in the CSF of injured mice and with an increased number of cells expressing TGF-α in the ipsilateral SVZ and in the injured cortex as soon as 7 dpi. The phenotype of these TGF-α ^+^ cells was mainly microglial cells in both the SVZ and injured cortex. Our results show that in response to the injury, Iba1^+^ microglial cells appear in the perilesional area and the SVZ. These microglial cells expressed TGF-α. Similar results have previously been found by Goings et al. Using aspiration lesions in the cerebral cortex of mice, they observe the presence of Iba1^+^ microglial cells in the cortex, striatum and corpus callosum of injured mice [58]. TGF-α expression has been found in injured regions in other cell types such as Nestin^+^ cells and astroglial (GFAP^+^) cells [11].

The elevated expression of TGF- α is accompanied by an overexpression of its receptor EGFR. Recent reports show that TGF-α expression is significantly increased in microglia/macrophages and neurons after ischemia. Likewise, the expression of the TGF-α receptor, EGFR is also increased [59]. In order to understand whether the increase in TGF- α played a role in the regulation of post injury neurogenesis, we started by evaluating neurogenesis in the SVZ. Accumulated evidences show that EGFR in the SVZ stimulates the activation of NSC, the proliferation of TAC [36–38] and the proliferation of immature neuroblasts [33]. Our results show that in the ipsilateral SVZ of injured mice an increased number of Ki67^+^ proliferating cells is found together with an elevated number of EGFR^+^ cells. These results indicate that, according to previous reports, [11, 14, 18], a proliferative response to the injury is found in the SVZ of injured mice that is concomitant with the increase in the number of EGFR^+^ cells, with the upregulation of the EGFR and TGF-α mRNAs, and with an elevated concentration of TGF-α in the CSF. An upregulation of the mRNA expression of other EGFR ligands, such as EGF, has previously been shown around the lesion and extending into the SVZ [16]. This finding supported the idea of the role that the EGFR-initiated pathway plays in neurogenesis post-injury. This is the first time in which an increased concentration of TGF-α in the CSF is described in response to a cortical injury. Interestingly, a higher number of neuroblasts within the ipsilateral SVZ of injured mice showed an immature phenotype in response to the injury, expressing EGFR and the cell cycle marker Ki67 suggesting that the stimulation of the EGFR-initiated pathway may impair maturation. This hypothesis was demonstrated by the fact that the inhibition of TGF-α release using a cPKC inhibitor, dramatically reduced the concentration of TGF-α in the CSF as well as the proportion of immature proliferating neuroblasts in the ipsilateral SVZ compared to the contralateral. We did not observe changes in the total pool of DCX^+^ cells. We explain these results considering that the pool of neuroblasts in the SVZ at any given point is a consequence of the addition of the number of generated neuroblasts minus the neuroblasts that have migrated to the OB. In mice that have not been treated with the cPKC inhibitor, the excess of TGF-α would increase proliferation of NSC, and EGFR expressing neuroblasts, while delaying OB migration. On the contrary, the treatment with the cPKC inhibitor would favor differentiation of undifferentiated progenitors into neuroblasts, and it would facilitate migration to the OB. The cPKC-dependent release of TGF-α has previously been demonstrated in cell cultures using time lapse images and fluorescent probes [20, 39, 40]. In here we observe a similar effect of the cPKC inhibitor in vivo when administered intranasally*,* that is accompanied by a reduction in the percentage of immature neuroblasts in the SVZ. Accordingly, a previous report shows that immature EGFR^+^ proliferating neuroblasts are found in the SVZ of mice transfected with a vector expressing TGF-α. In these mice, migration of the immature neuroblasts toward the OB is impaired [33].

Finally, in order to demonstrate the role of TGF-α and EGFR on the migration of neuroblasts from the SVZ toward brain injuries we have treated adult mice, in which we performed mechanical cortical injuries, with the EGFR inhibitor Afatinib; using a lentiviral vector that expressed the green fluorescent protein ZsGreen, we show that the inhibition of EGFR facilitates the enrichment of ZsGreen^+^ neuroblast around the perilesional area. Imaging techniques show that these neuroblasts migrate in chains from the SVZ towards the injured cortex. Interestingly, a previous report from our laboratory shows that when PKC activity is inhibited locally within the injury, an enrichment in neuroblasts can be observed. However, these neuroblasts do not seem to be SVZ-migrating cells [18]. Thus, it is possible that TGF-α exerts its antimigratory effect on SVZ neuroblasts and that Afatinib administered intranasally reaches the SVZ and exerts its effect on SVZ neuroblasts. Interestingly, a higher number of neuroblasts was found in the contralateral OB of Afatinib treated mice compared to non-treated mice. These results, which highlight the role of the signaling pathway TGF-α /EGFR as inhibitor of neuroblast migration agrees with a previous work, which shows that an excess TGF-α in the SVZ, impairs emigration of neuroblast toward the OB [33].

Afatinib is a tyrosine kinase inhibitor, which use was approved by the FDA in 2013 [60] for the treatment of patients with locally advanced or metastatic NSCLC with activating EGFR mutations who are EGFR tyrosine kinase inhibitor naïve [61]. We have chosen Afatinib to inhibit EGFR in this work, however, we know that other receptors of the ErbB family could be lightly inhibited by Afatinib. Alternatively, other EGFR inhibitors could be of use such as erlotinib or gefitinib, and testing a battery of these inhibitors may be interesting in future studies.

**Fig. 8:**
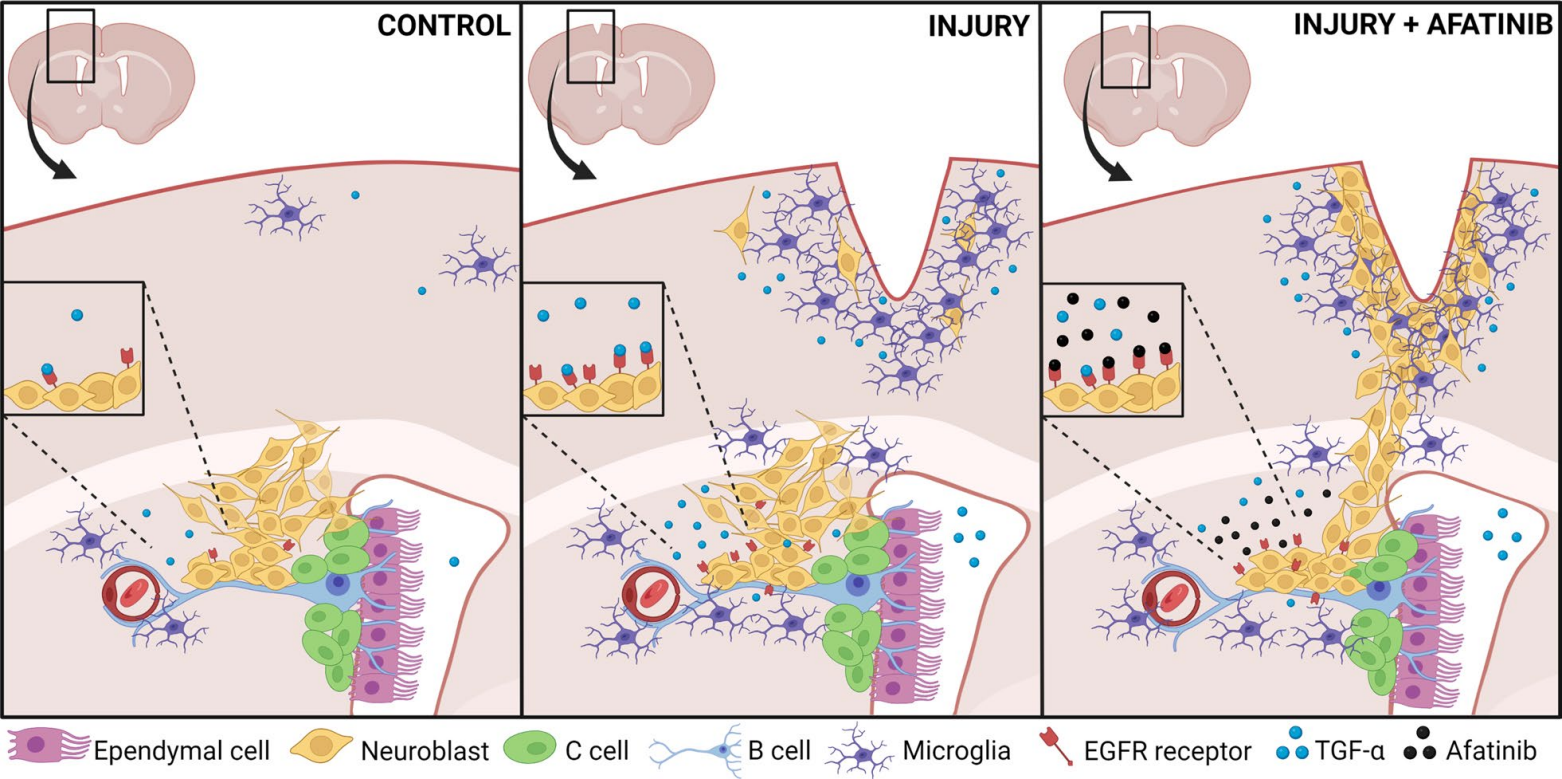
Effect of a cortical injury on TGF-α release and neuroblast migration. Drawing represents microglial cells and neuroblasts in the SVZ and cortical area before the injury (left panel) in response to the injury (central panel) and after Afatinib treatment (right panel). The differential expression of TGF-α and EGFR in each condition is represented as well as TGF-α release. Image was created using Biorender (www.biorender.com)

Thus, as summarized in Fig. 8, we show in here that in response to the injury a large number of microglial cells expressing TGF-α emerge within the SVZ and the injured area. TGF-α is released from these cells and activates the EGFR present in the neuroblast membrane inducing its proliferation, delaying maturation and probably impairing migration. The use of the EGFR inhibitor Afatinib stimulates neuroblast migration and enrichment in neuroblasts of the injured area. These neuroblasts could probably differentiate into mature cortical neurons if the treatment persisted. Our results suggest that TGF-α or EGFR may be used as targets to regenerate brain injuries. The use of this or other EGFR inhibitors upon mechanical cortical injuries may result in the repair of the injured brain tissue.

### Supplementary Information

**Additional file 1**: **Figure S1**. Inhibition of EGFR does not change the number of SVZ ZSGreen+ neuroblasts in response to a cortical injury. **Figure S2**. Inhibition of EGFR does not change the number of OB ZSGreenneuroblasts in response to a cortical injury. **Table S1**. List of primary antibodies used in the study. Specifying host, isotype, dilution used, epitope retrieval, staining pattern, source and reference. **Table S2**. List of secondary antibodies used in the study. Specifying host, dilution used, fluorescence conjugated, source and reference.

## Data Availability

All data in databases will be available to anyone upon request.

## References

[1] Toda T, Gage FH (2018). Review: adult neurogenesis contributes to hippocampal plasticity. Cell Tissue Res.

[2] Obernier K, Alvarez-Buylla A (2019). Neural stem cells: origin, heterogeneity and regulation in the adult mammalian brain. Development.

[3] Ceanga M, Dahab M, Witte OW, Keiner S (2021). Adult neurogenesis and stroke: a tale of two neurogenic niches. Front Neurosci.

[4] Alvarez-Buylla A, Garcia-Verdugo JM (2002). Neurogenesis in adult subventricular zone. J Neurosci.

[5] Goldman JE (2003). What are the characteristics of cycling cells in the adult central nervous system?. J Cell Biochem.

[6] Chaker Z, Codega P, Doetsch F (2016). A mosaic world: puzzles revealed by adult neural stem cell heterogeneity. Wiley Interdiscip Rev Dev Biol.

[7] Lim DA, Alvarez-Buylla A (2016). The adult ventricular-subventricular zone (V-SVZ) and Olfactory Bulb (OB) neurogenesis. Cold Spring Harb Perspect Biol.

[8] Llorens-Bobadilla E, Zhao S, Baser A, Saiz-Castro G, Zwadlo K, Martin-Villalba A (2015). Single-cell transcriptomics reveals a population of dormant neural stem cells that become activated upon brain injury. Cell Stem Cell.

[9] Jin K, Minami M, Lan JQ, Mao XO, Batteur S, Simon RP (2001). Neurogenesis in dentate subgranular zone and rostral subventricular zone after focal cerebral ischemia in the rat. Proc Natl Acad Sci U S A.

[10] Moraga A, Pradillo JM, Cuartero MI, Hernandez-Jimenez M, Oses M, Moro MA (2014). Toll-like receptor 4 modulates cell migration and cortical neurogenesis after focal cerebral ischemia. FASEB J.

[11] Romero-Grimaldi C, Murillo-Carretero M, Lopez-Toledano MA, Carrasco M, Castro C, Estrada C (2011). ADAM-17/tumor necrosis factor-alpha-converting enzyme inhibits neurogenesis and promotes gliogenesis from neural stem cells. Stem Cells.

[12] Arvidsson A, Collin T, Kirik D, Kokaia Z, Lindvall O (2002). Neuronal replacement from endogenous precursors in the adult brain after stroke. Nat Med.

[13] Hou SW, Wang YQ, Xu M, Shen DH, Wang JJ, Huang F (2008). Functional integration of newly generated neurons into striatum after cerebral ischemia in the adult rat brain. Stroke.

[14] Parent JM, Vexler ZS, Gong C, Derugin N, Ferriero DM (2002). Rat forebrain neurogenesis and striatal neuron replacement after focal stroke. Ann Neurol.

[15] Yamashita T, Ninomiya M, Hernandez Acosta P, Garcia-Verdugo JM, Sunabori T, Sakaguchi M (2006). Subventricular zone-derived neuroblasts migrate and differentiate into mature neurons in the post-stroke adult striatum. J Neurosci.

[16] Sundholm-Peters NL, Yang HK, Goings GE, Walker AS, Szele FG (2005). Subventricular zone neuroblasts emigrate toward cortical lesions. J Neuropathol Exp Neurol.

[17] Zhang RL, LeTourneau Y, Gregg SR, Wang Y, Toh Y, Robin AM (2007). Neuroblast division during migration toward the ischemic striatum: a study of dynamic migratory and proliferative characteristics of neuroblasts from the subventricular zone. J Neurosci.

[18] Garcia-Bernal F, Geribaldi-Doldan N, Dominguez-Garcia S, Carrasco M, Murillo-Carretero M, Delgado-Ariza A (2018). Protein kinase c inhibition mediates neuroblast enrichment in mechanical brain injuries. Front Cell Neurosci.

[19] Geribaldi-Doldan N, Carrasco M, Murillo-Carretero M, Dominguez-Garcia S, Garcia-Cozar FJ, Munoz-Miranda JP (2018). Specific inhibition of ADAM17/TACE promotes neurogenesis in the injured motor cortex. Cell Death Dis.

[20] Dominguez-Garcia S, Geribaldi-Doldan N, Gomez-Oliva R, Ruiz FA, Carrascal L, Bolivar J (2020). A novel PKC activating molecule promotes neuroblast differentiation and delivery of newborn neurons in brain injuries. Cell Death Dis.

[21] Magavi SS, Macklis JD (2002). Induction of neuronal type-specific neurogenesis in the cerebral cortex of adult mice: manipulation of neural precursors in situ. Brain Res Dev Brain Res.

[22] Buffo A, Rite I, Tripathi P, Lepier A, Colak D, Horn AP (2008). Origin and progeny of reactive gliosis: A source of multipotent cells in the injured brain. Proc Natl Acad Sci U S A.

[23] Nakatomi H, Kuriu T, Okabe S, Yamamoto S, Hatano O, Kawahara N (2002). Regeneration of hippocampal pyramidal neurons after ischemic brain injury by recruitment of endogenous neural progenitors. Cell.

[24] Saha B, Peron S, Murray K, Jaber M, Gaillard A (2013). Cortical lesion stimulates adult subventricular zone neural progenitor cell proliferation and migration to the site of injury. Stem Cell Res.

[25] Romero-Grimaldi C, Murillo-Carretero M, Angel Lopez-Toledano M, Carrasco M, Castro C, Estrada C (2011). ADAM-17/tumor necrosis factor-alpha-converting enzyme inhibits neurogenesis and promotes gliogenesis from neural stem cells. Stem Cells.

[26] Seidenfaden R, Desoeuvre A, Bosio A, Virard I, Cremer H (2006). Glial conversion of SVZ-derived committed neuronal precursors after ectopic grafting into the adult brain. Mol Cell Neurosci.

[27] Kawano H, Kimura-Kuroda J, Komuta Y, Yoshioka N, Li HP, Kawamura K (2012). Role of the lesion scar in the response to damage and repair of the central nervous system. Cell Tissue Res.

[28] Sofroniew MV, Vinters HV (2010). Astrocytes: biology and pathology. Acta Neuropathol.

[29] Carulli D, Laabs T, Geller HM, Fawcett JW (2005). Chondroitin sulfate proteoglycans in neural development and regeneration. Curr Opin Neurobiol.

[30] Fawcett JW, Asher RA (1999). The glial scar and central nervous system repair. Brain Res Bull.

[31] Silver J, Miller JH (2004). Regeneration beyond the glial scar. Nat Rev Neurosci.

[32] Jassam YN, Izzy S, Whalen M, McGavern DB, El Khoury J (2017). Neuroimmunology of traumatic brain injury: time for a paradigm shift. Neuron.

[33] Kim Y, Comte I, Szabo G, Hockberger P, Szele FG (2009). Adult mouse subventricular zone stem and progenitor cells are sessile and epidermal growth factor receptor negatively regulates neuroblast migration. PLoS ONE.

[34] Ghashghaei HT, Weber J, Pevny L, Schmid R, Schwab MH, Lloyd KC (2006). The role of neuregulin-ErbB4 interactions on the proliferation and organization of cells in the subventricular zone. Proc Natl Acad Sci U S A.

[35] Marquardt H, Hunkapiller MW, Hood LE, Todaro GJ (1984). Rat transforming growth factor type 1: structure and relation to epidermal growth factor. Science.

[36] Pastrana E, Cheng LC, Doetsch F (2009). Simultaneous prospective purification of adult subventricular zone neural stem cells and their progeny. Proc Natl Acad Sci U S A.

[37] Cesetti T, Obernier K, Bengtson CP, Fila T, Mandl C, Holzl-Wenig G (2009). Analysis of stem cell lineage progression in the neonatal subventricular zone identifies EGFR+/NG2- cells as transit-amplifying precursors. Stem Cells.

[38] Codega P, Silva-Vargas V, Paul A, Maldonado-Soto AR, Deleo AM, Pastrana E (2014). Prospective identification and purification of quiescent adult neural stem cells from their in vivo niche. Neuron.

[39] Dang M, Armbruster N, Miller MA, Cermeno E, Hartmann M, Bell GW (2013). Regulated ADAM17-dependent EGF family ligand release by substrate-selecting signaling pathways. Proc Natl Acad Sci U S A.

[40] Dang M, Dubbin K, D'Aiello A, Hartmann M, Lodish H, Herrlich A (2011). Epidermal growth factor (EGF) ligand release by substrate-specific a disintegrin and metalloproteases (ADAMs) involves different protein kinase C (PKC) isoenzymes depending on the stimulus. J Biol Chem.

[41] McGrath JC, Drummond GB, McLachlan EM, Kilkenny C, Wainwright CL (2010). Guidelines for reporting experiments involving animals: the ARRIVE guidelines. Br J Pharmacol.

[42] Kilkenny C, Browne W, Cuthill IC, Emerson M, Altman DG, Group NCRRGW (2010). Animal research: reporting in vivo experiments: the ARRIVE guidelines. Br J Pharmacol.

[43] Rabaneda LG, Geribaldi-Doldan N, Murillo-Carretero M, Carrasco M, Martinez-Salas JM, Verastegui C (2016). Altered regulation of the Spry2/Dyrk1A/PP2A triad by homocysteine impairs neural progenitor cell proliferation. Biochim Biophys Acta.

[44] Murillo-Carretero M, Geribaldi-Doldan N, Flores-Giubi E, Garcia-Bernal F, Navarro-Quiroz EA, Carrasco M (2017). ELAC (3,12-di-O-acetyl-8-O-tigloilingol), a plant-derived lathyrane diterpene, induces subventricular zone neural progenitor cell proliferation through PKCbeta activation. Br J Pharmacol.

[45] Carrasco M, Rabaneda LG, Murillo-Carretero M, Ortega-Martinez S, Martinez-Chantar ML, Woodhoo A (2014). Glycine N-methyltransferase expression in the hippocampus and its role in neurogenesis and cognitive performance. Hippocampus.

[46] Thorne RG, Pronk GJ, Padmanabhan V, Frey WH (2004). Delivery of insulin-like growth factor-I to the rat brain and spinal cord along olfactory and trigeminal pathways following intranasal administration. Neuroscience.

[47] Marks DR, Tucker K, Cavallin MA, Mast TG, Fadool DA (2009). Awake intranasal insulin delivery modifies protein complexes and alters memory, anxiety, and olfactory behaviors. J Neurosci.

[48] Lim NKH, Moestrup V, Zhang X, Wang WA, Moller A, Huang FD (2018). An improved method for collection of cerebrospinal fluid from anesthetized mice. J Vis Exp.

[49] Geribaldi-Doldan N, Flores-Giubi E, Murillo-Carretero M, Garcia-Bernal F, Carrasco M, Macias-Sanchez AJ, Domínguez-Riscart J, Verástegui C, Hernández-Galán R, Castro C (2015). 12-deoxyphorbols promote adult neurogenesis by inducing neural progenitor cell proliferation via PKC activation. Int J Neuropsychopharmacol.

[50] Rabaneda LG, Carrasco M, Lopez-Toledano MA, Murillo-Carretero M, Ruiz FA, Estrada C (2008). Homocysteine inhibits proliferation of neuronal precursors in the mouse adult brain by impairing the basic fibroblast growth factor signaling cascade and reducing extracellular regulated kinase 1/2-dependent cyclin E expression. FASEB J.

[51] Ezzanad A, Gomez-Oliva R, Escobar-Montano F, Diez-Salguero M, Geribaldi-Doldan N, Dominguez-Garcia S (2021). Phorbol diesters and 12-deoxy-16-hydroxyphorbol 13,16-diesters induce TGFalpha release and adult mouse neurogenesis. J Med Chem.

[52] Curtis MJ, Abernethy DR (2015). Revision of instructions to authors for pharmacology research and perspectives: enhancing the quality and transparency of published work. Pharmacol Res Perspect.

[53] Geribaldi-Doldan N, Carrascal L, Perez-Garcia P, Oliva-Montero JM, Pardillo-Diaz R, Dominguez-Garcia S (2023). Migratory response of cells in neurogenic niches to neuronal death: the onset of harmonic repair?. Int J Mol Sci.

[54] Pencea V, Bingaman KD, Wiegand SJ, Luskin MB (2001). Infusion of brain-derived neurotrophic factor into the lateral ventricle of the adult rat leads to new neurons in the parenchyma of the striatum, septum, thalamus, and hypothalamus. J Neurosci.

[55] Nemirovich-Danchenko NM, Khodanovich MY (2019). New neurons in the post-ischemic and injured brain: migrating or resident?. Front Neurosci.

[56] Dayer AG, Cleaver KM, Abouantoun T, Cameron HA (2005). New GABAergic interneurons in the adult neocortex and striatum are generated from different precursors. J Cell Biol.

[57] Magnusson JP, Goritz C, Tatarishvili J, Dias DO, Smith EM, Lindvall O (2014). A latent neurogenic program in astrocytes regulated by Notch signaling in the mouse. Science.

[58] Goings GE, Kozlowski DA, Szele FG (2006). Differential activation of microglia in neurogenic versus non-neurogenic regions of the forebrain. Glia.

[59] Dai X, Chen J, Xu F, Zhao J, Cai W, Sun Z (2020). TGFalpha preserves oligodendrocyte lineage cells and improves white matter integrity after cerebral ischemia. J Cereb Blood Flow Metab.

[60] Dungo RT, Keating GM (2013). Afatinib: First Global Approval. Drugs.

[61] de Marinis F, Laktionov KK, Poltoratskiy A, Egorova I, Hochmair M, Passaro A (2021). Afatinib in EGFR TKI-naïve patients with locally advanced or metastatic EGFR mutation-positive non-small cell lung cancer: interim analysis of a Phase 3b study. Lung Cancer.

