# Supplementary material for: Targeting epidermal growth factor receptor to recruit newly generated neuroblasts in cortical brain injuries
Source: J Transl Med. 2023 Nov 30;21:867. doi: 10.1186/s12967-023-04707-1 (PMC10687845; doi:10.1186/s12967-023-04707-1)
Supplement: Supplementary file 1 — Additional file 1: Figure S1. Inhibition of EGFR does not change the number of SVZ ZSGreen+ neuroblasts in response to a cortical injury. Figure S2. Inhibition of EGFR does not change the number of OB ZSGreenneuroblasts in response to a cortical injury. Table S1. List of primary antibodies used in the study. Specifying host, isotype, dilution used, epitope retrieval, staining pattern, source and reference. Table S2. List of secondary antibodies used in the study. Specifying host, dilution used, fluorescence conjugated, source and reference. [file 12967_2023_4707_MOESM1_ESM.pdf]

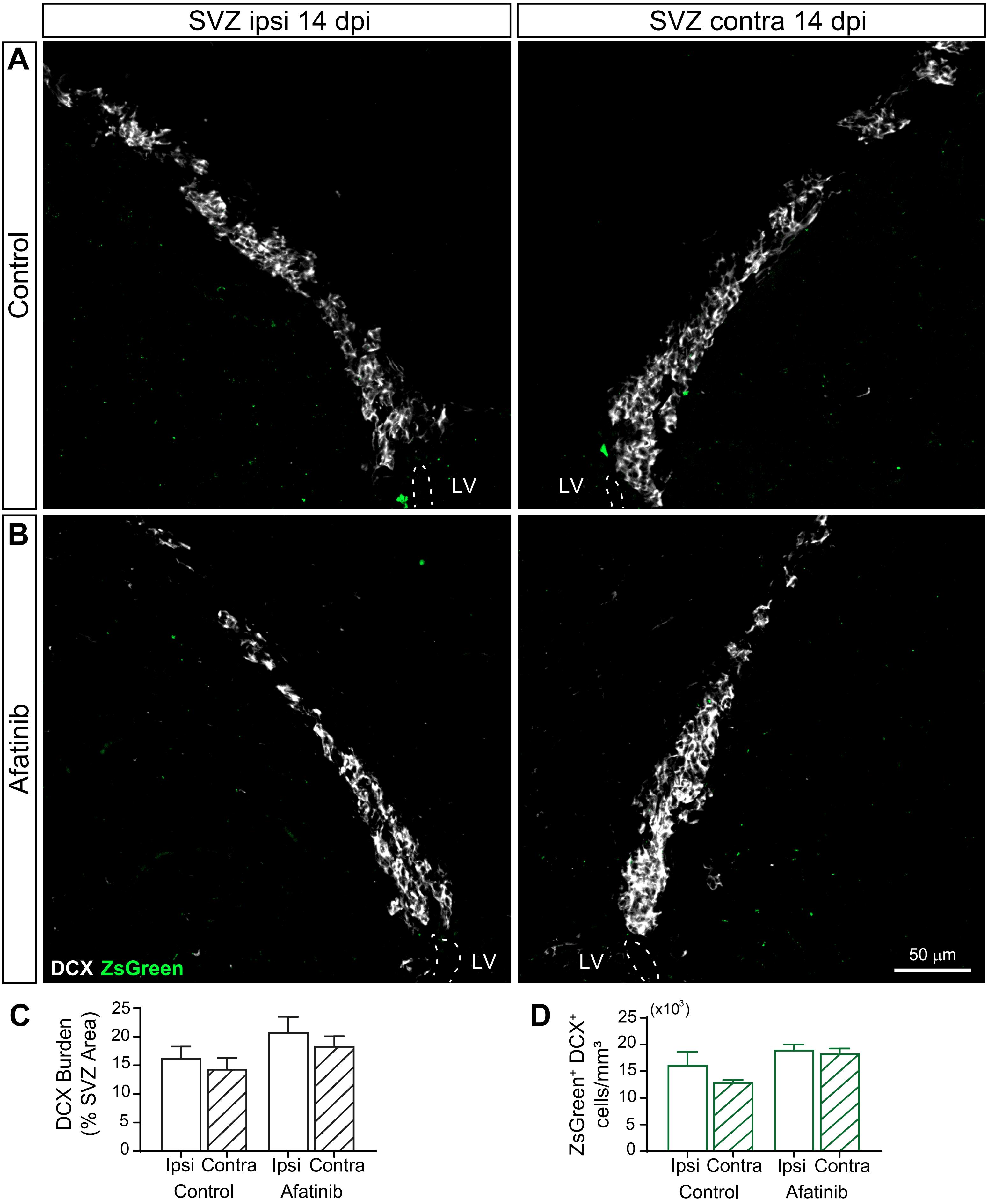

Additional file 1: Figure S1. Inhibition of EGFR does not change the number of SVZ ZSGreen$^+$ neuroblasts in response to a cortical injury. Mice were mechanically injured in the primary motor cortex and were injected in the lateral ventricle (LV) with a control lentivirus that express the green fluorescent protein ZsGreen. Mice were treated for 14 days with intranasal administrations of the EGFR inhibitor Afatinib (5 μM) or vehicle. **A.** Representative confocal images of adult mouse injured cortex processed for the detection of DCX and ZsGreen treated only with vehicle. **B.** Representative confocal images adult mouse injured cortex treated with Afatinib showing ZsGreen fluorescence and the immunodetection of DCX. Scale bar represents 50 μm. **C.** Graph represents the DCX burden as a percentage of SVZ total area. **D.** Graph represents the number of ZsGreen$^+$ and DCX$^+$ cells in the SVZ. One-way ANOVA was used for statistical analysis. Data are the mean ± S.E.M; n=6 animals per group.

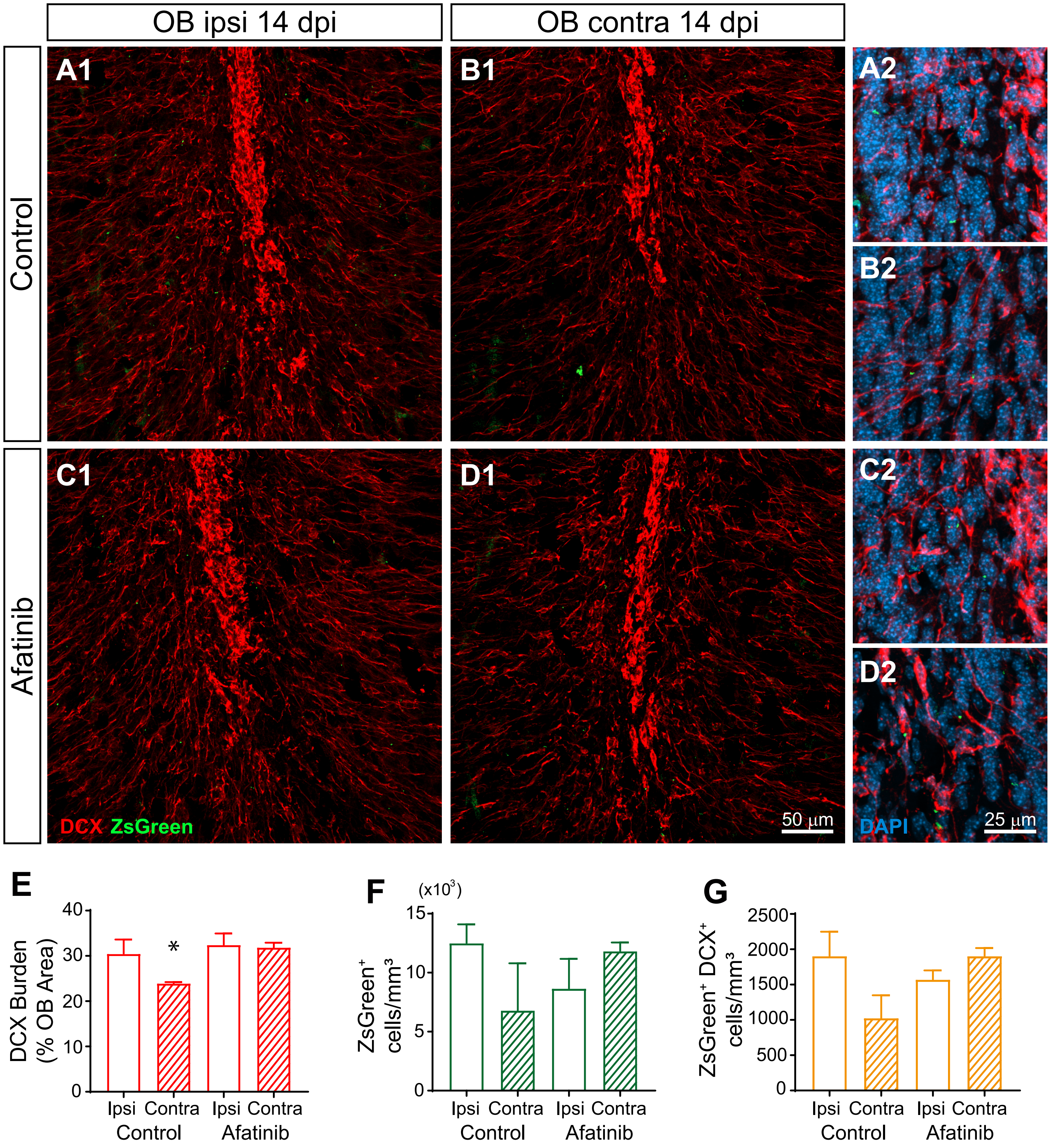

Additional file 1: Figure S2. Inhibition of EGFR does not change the number of OB ZSGreen$^+$ neuroblasts in response to a cortical injury. Mice were mechanically injured in the primary motor cortex and were injected in the lateral ventricle (LV) with a control lentivirus that express the green fluorescent protein ZsGreen. Mice were treated for 14 days with intranasal administrations of the EGFR inhibitor Afatinib (5 µM) or vehicle. **A1, B1.** Representative confocal images of adult mouse injured cortex processed for the detection of DCX and ZsGreen treated only with vehicle. Magnification of representative cells showing DCX$^+$ ZsGreen$^+$ DAPI$^+$ cells are represented in **A2** and **B2**. **C1, D1.** Representative confocal images adult mouse injured cortex treated with Afatinib showing ZsGreen fluorescence and the immunodetection of DCX. Magnification of representative cells showing DCX$^+$ ZsGreen$^+$ DAPI$^+$ cells are represented in **C2** and **D2**. Scale bar represents 50 µm. **E.** Graph represents the DCX burden as a percentage of OB total area. **F.** Graph represents the number of ZsGreen$^+$ cells in the OB. **G.** Graph represents the number of ZsGreen$^+$ and DCX$^+$ cells in the OB. One-way ANOVA was used for statistical analysis: * $p < 0.05$ each condition compared with contra control. Data are the mean ± S.E.M; n = 6 animals per group.

Antibody	Host	Isotype	Dilution	Epitope retrieval	Staining pattern	Source	Reference
Anti-DCX	Rabbit	polyclonal	1:750	DCX, neuroblast marker	cytoplasmic	Abcam (Cambridge, UK)	ab18723
Anti-DCX	Goat	polyclonal	1:200	DCX, neuroblast marker	cytoplasmic	Abcam (Cambridge, UK)	ab113435
Anti-EGFR	Sheep	polyclonal	1:200	EGFR, epidermic growth factor receptor	cytoplasmic	Merk Millipore (Billerica, Ma, USA)	06-847
Anti-Iba1	Rabbit	monoclonal	1:500	Iba1, microglia marker	cytoplasmatic	Abcam (Cambridge, UK)	ab178846
Anti-Ki67	Rabbit	polyclonal	1:500	Ki67, proliferation marker	nuclear	Abcam (Cambridge, UK)	ab15580
Anti-TGFα	Mouse	monoclonal	1:100	TGF-α, Transforming Growth Factor Alpha [illegible]	cytoplasmatic	Santa Cruz Biotechnology (Santa [illegible]	sc-374433

Additional file 1: Table S1. List of primary antibodies used in the study. Specifying host, isotype, dilution used, epitope retrieval, staining pattern, source and reference.

Antibody	Host	Dilution	Fluorescence	Source	Reference
Alexa Flour anti-rabbit	Donkey	1:1000	647	Invitrogen (Carlsbad, CA, USA)	A32795
Alexa Flour anti-mouse	Donkey	1:1000	488	Invitrogen (Carlsbad, CA, USA)	A-21206
Alexa Flour anti-mouse	Donkey	1:1000	594	Invitrogen (Carlsbad, CA, USA)	A-21203
Alexa Flour anti-sheep	Donkey	1:1000	594	Invitrogen (Carlsbad, CA, USA)	A-11016
Alexa Flour anti-rabbit	Donkey	1:1000	594	Invitrogen (Carlsbad, CA, USA)	A-21207
Alexa Flour anti-rabbit	Donkey	1:1000	488	Invitrogen (Carlsbad, CA, USA)	A-21206
Alexa Flour -anti-goat	Donkey	1:1000	594	Invitrogen (Carlsbad, CA, USA)	A-11058

Additional file 1: Table S2. Secondary antibodies supplementary table 2: List of secondary antibodies used in the study. Specifying host, dilution used, fluorescence conjugated, source and reference.
